# Supplementary material for: Ependymal cilia beating induces an actin network to protect centrioles against shear stress
Source: Nat Commun. 2018 Jun 11;9:2279. doi: 10.1038/s41467-018-04676-w (PMC5996024; doi:10.1038/s41467-018-04676-w)
Supplement: Supplementary file 1 — Supplementary Information [file 41467_2018_4676_MOESM1_ESM.pdf]

Ependymal cilia beating induces an actin network to protect centrioles against shear stress

Mahuzier et al.

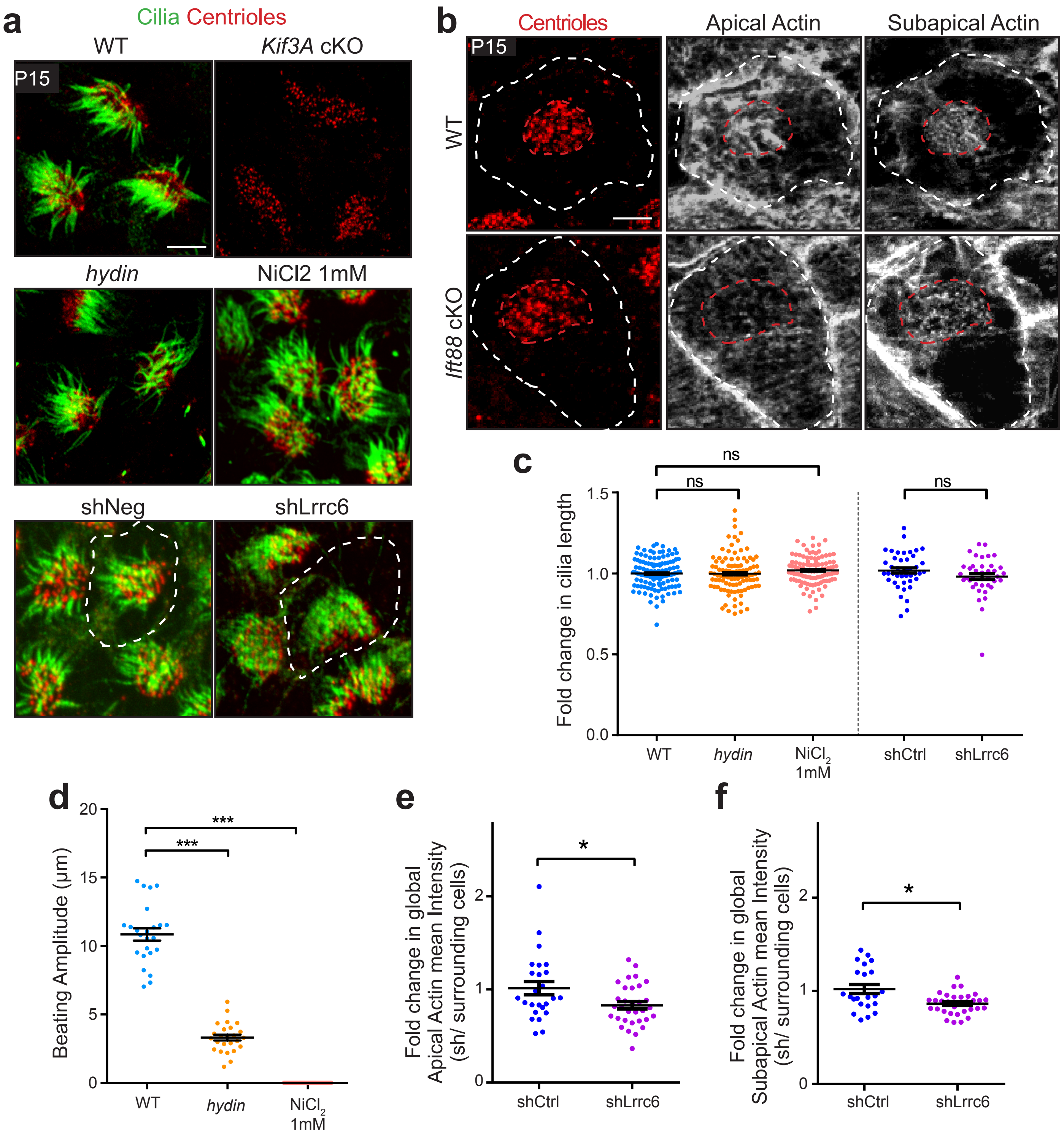

Supplementary Fig. 1: Cilia and actin in ciliary defective mice.
a, Mature ependymal cells on lateral ventricular walls of P15 mice, mutant for ciliary genes (*Kif3A-cKO* or *hydin*) or WT-untreated, -treated for 40h with 1mM $NiCl_2$ or -transfected at birth with shCtrl or shLrrc6, in which cilia (GT335, green) and centrioles (FOP, red) were stained. Motile cilia are absent only in *Kif3A-cKO*. **b,** F-actin (phalloidin, gray) and centrioles (FOP, red) in mature ependymal cells (P15) in control (WT) and the ciliary mutant mice *Ift88-cKO*; cell borders and centriolar patches are outlined with dashed white and red lines, respectively. Apical actin enriched in the centriolar patch is reduced in ciliary mutants. **c,** Fold change in cilia length of ependymal cells of *hydin* ciliary mutants relative to littermate controls or explants treated with $NiCl_2$ relative to the contralateral wall treated with PBS or ependymal cells transfected with shRNA (shCtrl or shLrrc6) relative to surrounding un-transfected cells; cilia length is not affected by the impairment of cilia motility. n=130 cells for WT, 101 for *hydin* mutants and 106 from $NiCl_2$-treated explants; p-values were determined by one-way ANOVA followed by Dunn's multiple comparison test; ns, P>0.05. **d,** Amplitude of cilia beating (µm) measured on motile cilia in brain slices under the indicated conditions. n=25 cells for WT, *hydin* mutants and $NiCl_2$-treated explants; p-values were determined with the Mann-Whitney test; *** P≤0.0001. **(e, f)** Mean phalloidin fluorescence intensities inside the border of transfected cells relative to the mean of the three closest none-transfected control cells at the apical **(e)** and subapical **(f)** levels; apical and subapical actin networks decrease in Lrrc6-depleted cells. At the apical and subapical level, n=22 cells for shCtrl and 32 for shLrrc6 transfected cells. p-values were determined by the Mann Whitney test; * P=0.03 and 0.01 for the apical and subapical levels, respectively. Scale bars, 5 µm. Error bar represent the sem between n of three independent experiments in all graphs.

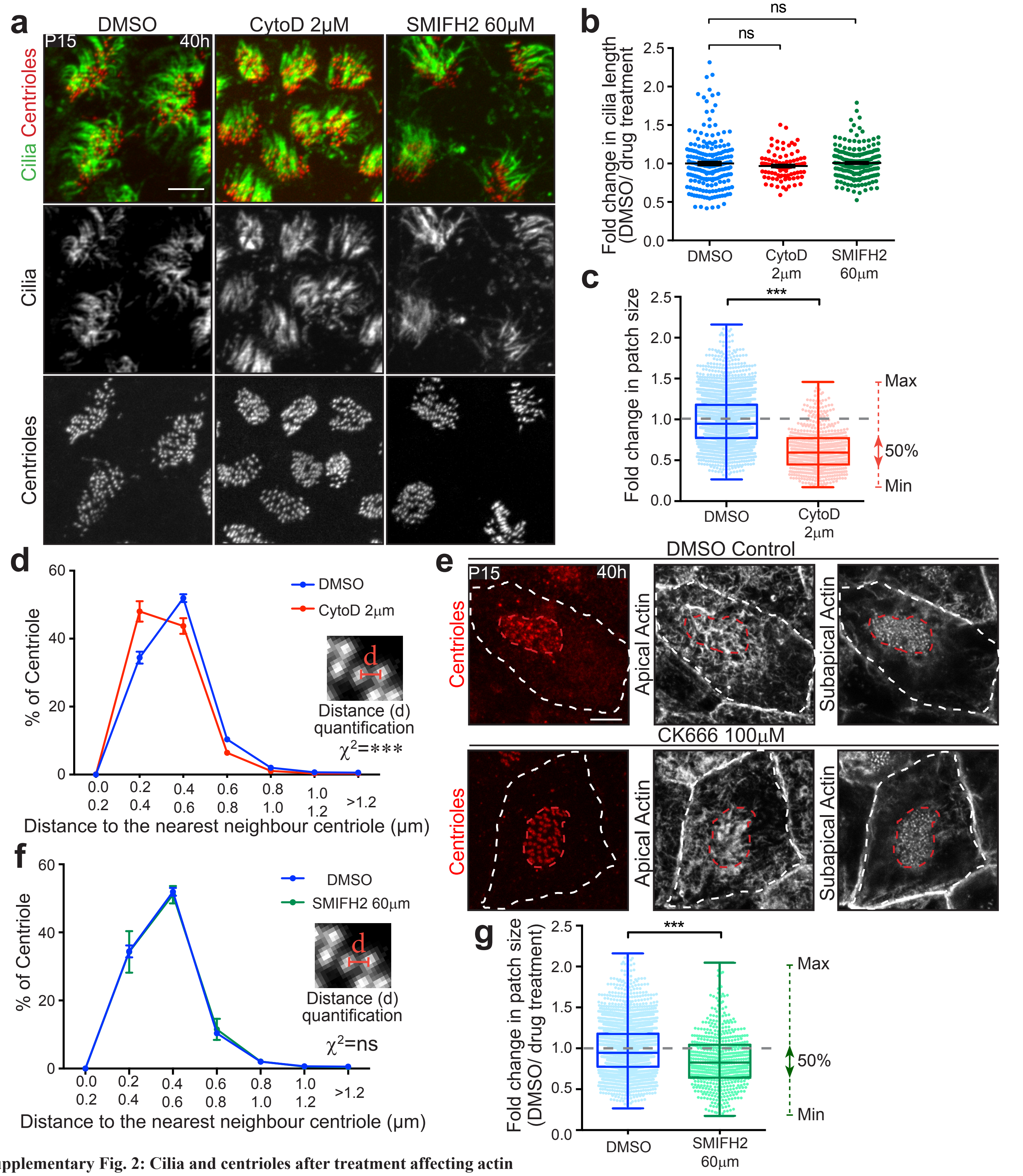

Supplementary Fig. 2: Cilia and centrioles after treatment affecting actin

a, Cilia (GT335, green) and centrioles (FOP, red) staining on mature (P15) ependymal cell lateral ventricular wall explants after a 40h treatment with DMSO (controls), cytochalasin-D (CytoD 2μM) or the formin inhibitor SMIFH2 (60μM). **b,** Fold change in cilia length in DMSO-, CytoD- or SMIFH2-treated explants, relative to the contralateral wall treated with DMSO. n=206 cells for DMSO-, 194 for SMIFH2- and 91 for CytoD-treated explants; p-values were determined by one-way ANOVA followed by Dunn's multiple comparison test. **c,** Fold change in centriolar patch size in explants of Centrin2-GFP mice treated with DMSO (controls) or cytochalasin-D (2μM) for 40h, relative to contralateral wall explant treated with DMSO; patch size decreases after treatment with cytochalasin-D; p-values were determined with the Mann-Whitney test. n=2411 cells for DMSO- and 707 from CytoD-treated explants. **d,** Quantification of the distance of each centriole from its nearest neighbour in explants from P15 Centrin2-GFP mice treated with DMSO or cytochalasin-D (2μM) for 40h. n=136146 centriolar distance for DMSO- and 37285 for CytoD-treated explants; p-values were determined with the Chi^2 test for trend. **e,** F-actin (phalloidin, gray) and centrioles (FOP, red) in mature ependymal cells from explants treated with DMSO or the Arp2/3 inhibitor CK666 (100μM). Cell borders and centriolar patches are outlined with dashed white and red lines, respectively. No differences in actin organisation were noted. **f,** Quantification of the distance of each centriole from its nearest neighbour in explants from P15 Centrin2-GFP mice treated with DMSO or SMIFH2 for 40h. n>29701 centrioles; p-values were determined with the Chi^2 test for trend. **g,** Fold change in centriolar patch size in explants of Centrin2-GFP mice treated with DMSO (controls) or SMIFH2 for 40h, relative to the contralateral wall explant DMSO-treated; patch size decreases after treatment with SMIFH2; p-values were determined with the Mann-Whitney test. n=2411 cells for DMSO-and 601 for SMIFH2-treated explants. Scale bar, 5μm. Error bar represent the sem between n of three independent experiments in dot plot graphs or between the three independent experiments in the distribution graphs ns, P>0.05.*** P≤0.0001.

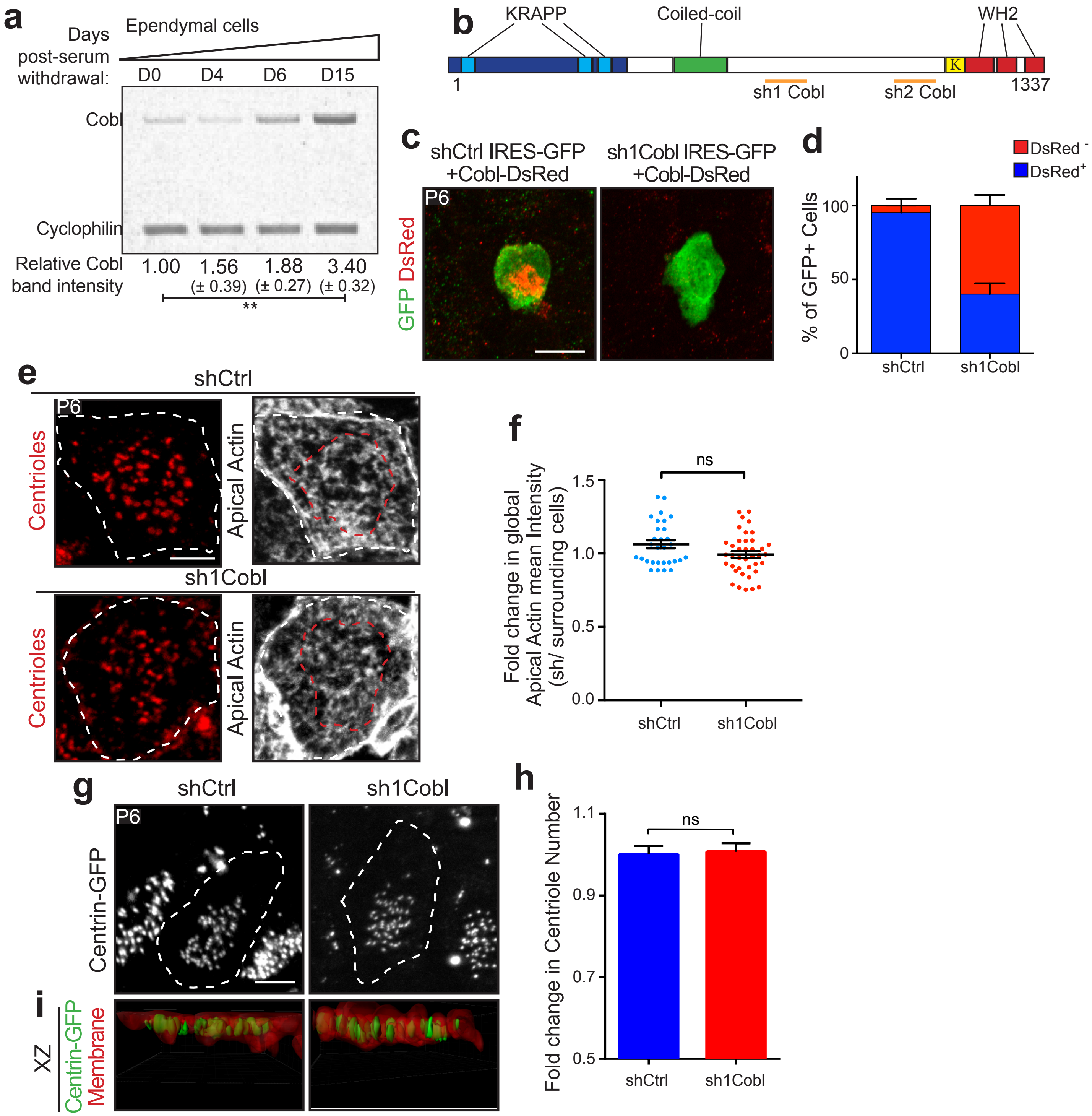

Supplementary Fig. 3: Cobl-expression and -depletion at early stages of differentiation.

a, Representative semi-quantitative RT-PCR on Cobl mRNA extracted from ependymal cells at different stages of differentiation: D0, progenitor cells; D4 and 6, differentiating ependymal cells; D15, mature ependymal cells. Intensities of Cobl- normalised with cyclophilin-DNA bands are represented as the mean±sem. p-values were determined with the Mann-Whitney test on three independent experiments; ** P=0.0018. **b,** Mouse Cobl domains: 'KRAPP', proline-rich ; WH2, Wiskott-Aldrich syndrome protein-homology2; K, lysine-rich and regions targeted by shRNA (sh1Cobl and sh2Cobl). **(c,d),** Cobl-depletion in cells co-transfected with shCtrl-IRES-GFP or sh1Cobl-IRES-GFP (GFP, green) and COBL-Ds-Red (dsRed, Red). **c,** Representative images. **d,** Percentage of GFP+ cells co-expressing Cobl-dsRed. n>30 cells. **e,** F-actin (phalloidin, gray) and centrioles (FOP, red) in lateral walls of immature ependymal cells (P6) from mice transfected at birth with shCtrl or sh1Cobl and a reporter (mCherry, white dashed lines); the apical actin network was not disrupted in immature ependymal cells. Note the absence of actin enrichment at the centriolar patches and subapical networks in both control and Cobl-depleted cells at this stage; red dashed lines define the centriolar patches. **f,** Apical actin (phalloidin) mean fluorescence intensities within the cell border of immature ependymal cells relative to that in the surrounding cells. n=30 cells for shCtrl and 39 for sh1Cobl transfected cells. **g,** Immature ependymal cells from the lateral ventricular wall of P6 Centrin2-GFP mice electroporated with shCtrl or sh1Cobl (dashed lines) at birth. **h,** Fold change in centriole number in sh-transfected cells relative to surrounding un-transfected cells; the number of centrioles did not decrease in immature Cobl-depleted ependymal cells. n=72 cells for shCtrl and sh1Cobl transfected cells. **i,** 3D-modelling of an XZ projection of centrioles (Green) embedded in the plasma membrane (mCherry reporter, red) in immature (P6) ependymal cells after electroporation with shCtrl or sh1Cobl at birth; the migration and docking of centrioles in Cobl-depleted cells is normal. Scale bars, 5 µm. In all graphs, error bar represent the sem between n of three independent experiments. p-values were determined with the Mann-Whitney test; ns, P>0.05.

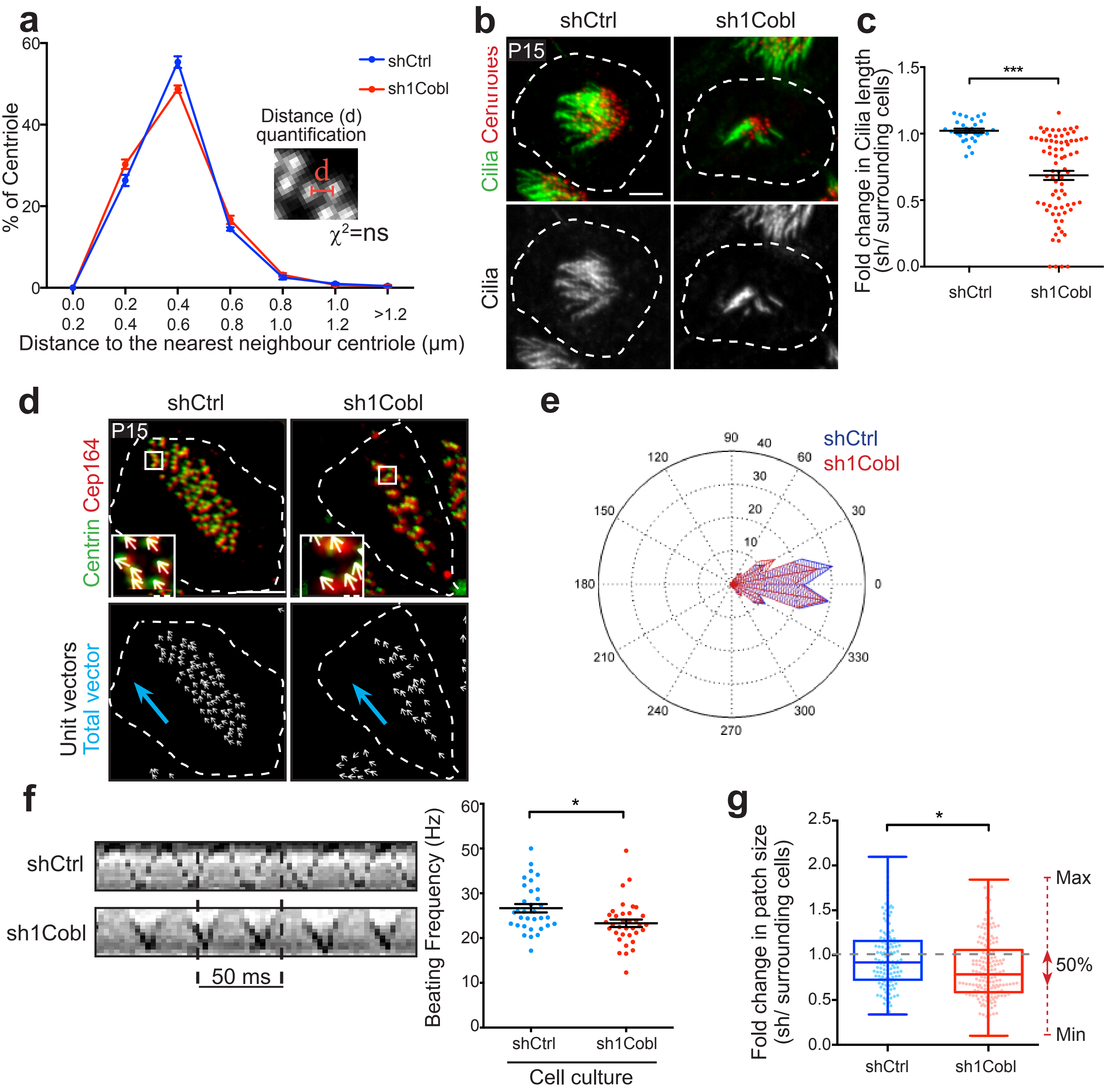

Supplementary Fig. 4: Cobl depletion at late stage of ependymal differentiation.
(a-e), Lateral ventricular walls of wild-type or Centrin2-GFP transgenic mice electroporated at birth with shCtrl or sh1Cobl and the mCherry-reporter construct (dashed lines). **a,** Distance between nearest neighbour centrioles in control (shCtrl) or Cobl-depleted (sh1Cobl) ependymal cells. n=4111 distances for shCtrl and 3526 for sh1Cobl cells. p-values were determined with the Chi^2 test for trend; ns, P>0.05. **b,** Mature Cen2-GFP$^+$ (Centriole, Red) ependymal cells stained for cilia (GT335, green), showing shorter cilia in Cobl-depleted cells compared to control. **c,** Fold change in cilia length in control (shCtrl) and Cobl-depleted ependymal cells (sh1Cobl) relative to surrounding un-transfected cells. n=30 cells for shCtrl and 77 for sh1Cobl cells. p-values were determined with the Mann-Whitney test; *** P≤0.0001. **d,** Vectors (white arrows) traced automatically between centrin2-GFP- (green) and Cep164-stained centrioles (red) showing centriole orientation in control (shCtrl) or Cobl-depleted (sh1Cobl) cells (dashed lines) . The global beating direction (blue arrows) of each cell is the mean of the individual vectors. **e,** Angles between each individual vector and the general beating vector showing that the orientation of centrioles was globally equivalent, although slightly more disperse, in Cobl-depleted cells compared to controls; p-values were determined with the Kolmogorov-Smirnov test (P=0.0006) and the Waston-U2 circular permutation (P=0.0008; 50,000 iterations) on n>65 cells from three independent experiments. **f,** Given the low efficiency of *in vivo* electroporation Cobl was depleted *in vitro*. Representative kymographs (200ms) of motile cilia in differentiated ependymal cell in culture (14 days after onset of differentiation); the ciliary beating frequency (Hz) in Cobl-depleted cells (sh1Cobl) was lower than in shCtrl. n=33 cells for shCtrl and 35 for sh1Cobl cells; p-values were determined by the Mann-Whitney test; ** P=0.0093. **g,** Fold change in centriolar patch size relative to neighbouring cells of P15 Centrin2-GFP transgenic mice electroporated at birth with shCtrl or sh1Cobl and the mCherry reporter construct, showing smaller patches in sh1Cobl compared to shCtrl cells. n=128 cells for shCtrl and 168 for sh1Cobl cells. p-values were determined with the Mann-Whitney test; ** P=0.0018. Scale bar, 5 μm. Error bar represent the sem between n of three independent experiments in dot plot graphs or between three independent experiments in the distribution graphs.

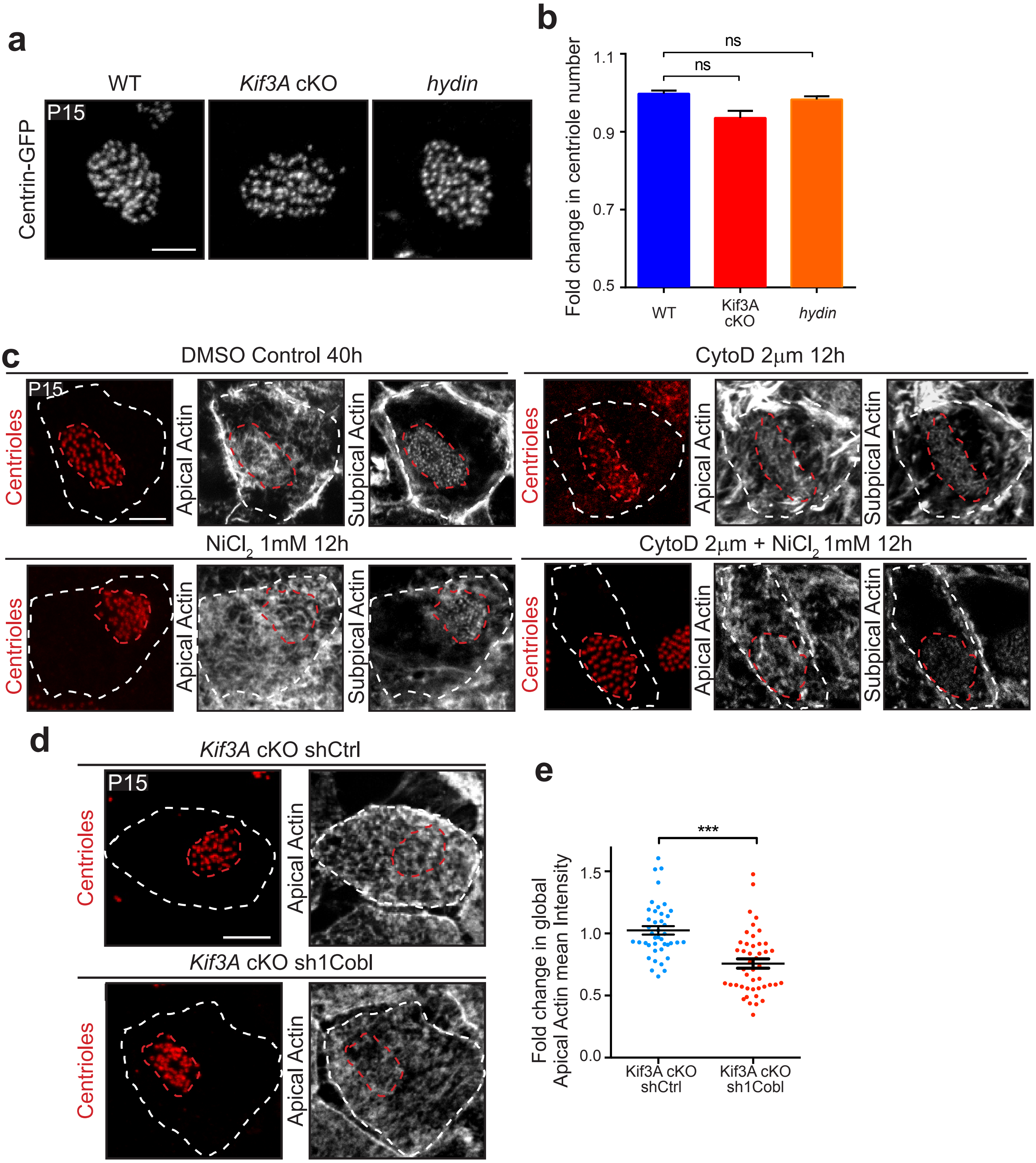

Supplementary Fig. 5: Ciliary motility, centriole and actin

a, Mature ependymal cells from Centrin2-GFP transgenic mice (P15): WT controls covered with motile cilia, *Kif3A-cKO* ciliary mutant devoid of motile cilia; *hydin* ciliary mutant covered with stiff cilia. **b,** Fold change in centriole number in control (WT) or ciliary mutant mice relative to the number of centrioles in littermate controls; control and mutant mice were similar; p-values were determined by one-way ANOVA followed by Dunn's multiple comparison test on n=604 cells for WT, 115 for *Kif3A cKO* and 588 for *hydin* mutants; ns, P>0.05. **c,** F-actin (phalloidin, gray) and centrioles (FOP, red) in mature ependymal cells from explants treated with DMSO or 2μM cytochalasin-D (CytoD) or 1mM $NiCl_2$ or both Cytochalasin-D and $NiCl_2$ for 12h showing similar actin impairment in cells treated with cytochalasin-D or with both cytochalasin-D and $NiCl_2$. **(d-e),** Lateral walls of ciliary mutant mice (*Kif3A cKO*) were electroporated at birth with shCtrl or sh1Cobl and a reporter construct (mCherry represented as dashed lines) and fixed at late stage of differentiation (P15 animals). **d,** F-actin (phalloidin, Gray) and centrioles (FOP, Red) of mature ependymal cells. **e,** Apical actin mean fluorescence intensities within the cell border in cells transfected with shCtrl or sh1Cobl relative to the surrounding un-transfected cells, showing a decrease of the apical actin in Cobl-depleted *Kif3A cKO* cells (*Kif3A-cKO* shCobl) relative to control *Kif3A-cKO* ependymal cells (*Kif3A-cKO* shCtrl). p-values were determined by the Mann-Whitney test; ***, P≤ 0.0001. n=42 cells for *Kif3A cKO* shCtrl and 45 for *Kif3A cKO* sh1Cobl transfected cells f. Scale bar, 5 μm. Error bar represent the sem between n of three independent experiments.

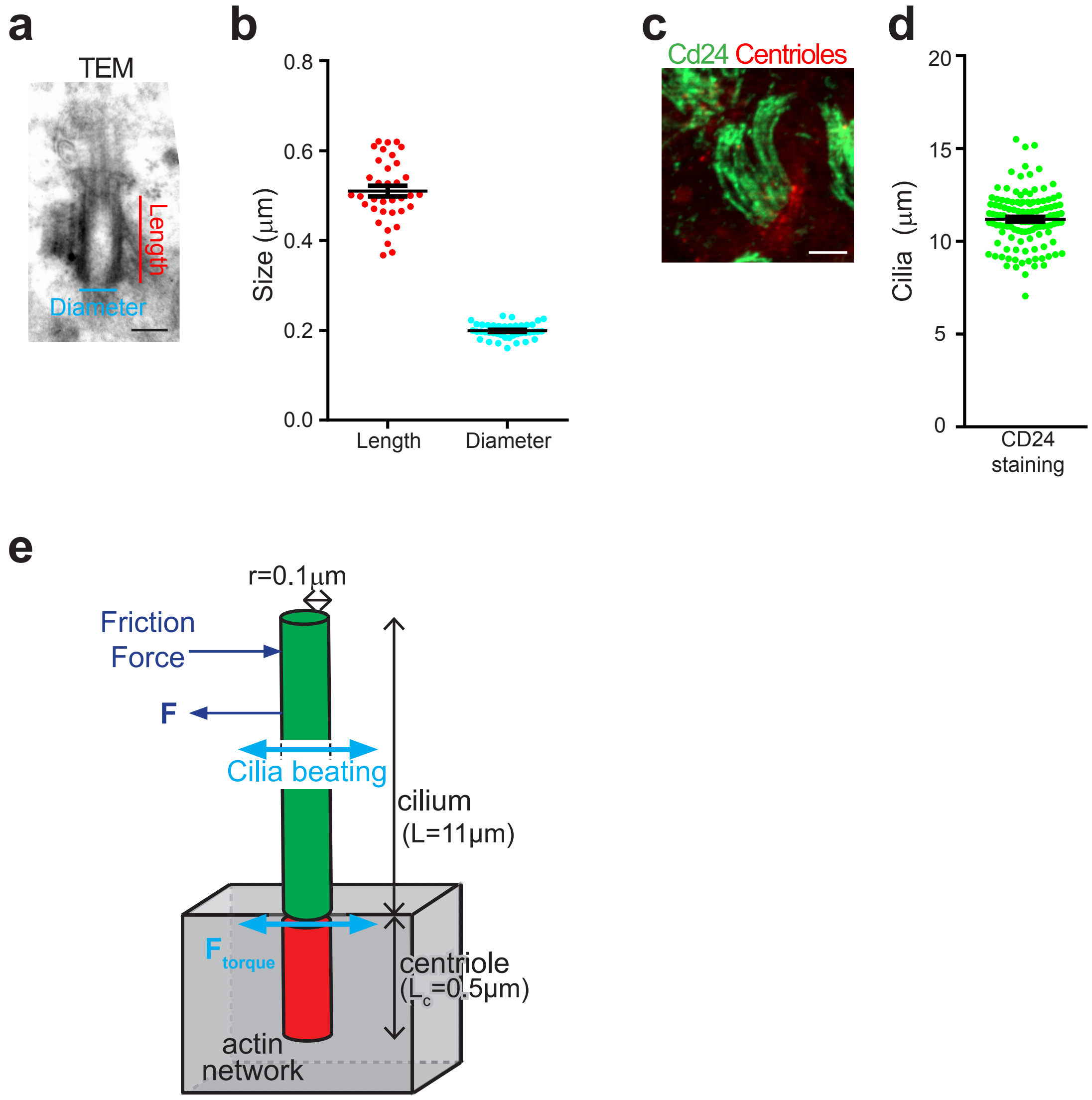

Supplementary Fig. 6: Geometrical characteristics of centrioles and motile cilia of ependymal cells

a, Transmission electron microscopy of centrioles of multiciliated ependymal cells at P21. Scale bar 0.2µm. **b,** Centriole diameter and length. Error bars represent the sem of n=43 centrioles for the diameter and 35 centrioles for the length. **c,** Ependymal cells at P15 stained for the membrane marker CD24 (cilia, green) and centrioles (20H5, Red). **d,** Cilia length evaluated on ependymal cells stained with CD24. Error bar represent the sem of n=122. **e,** Model of a motile cilium with its base embedded in an actin network: radius, r=99nm±2 (mean±sem) calculated on electron micrographs; cilia length, L=11.1µm±0.1 (mean±sem) measured using the ependymal cell surface marker CD24; centriole length, Lc=510nm±2 (mean±sem) calculated on electron micrographs. The hydrodynamic friction force exerted by the fluid flow and is resultant (F) are shown as dark blue arrows, while the additional torque exerted by cilia beating and its resultant (F_{torque}) are shown as light blue arrows;
